# Management of Acute Coronary Syndrome in Severe Diffuse Coronary Artery Ectasia: A Therapeutic Challenge

**DOI:** 10.1016/j.jaccas.2026.110203

**Published:** 2026-09-11

**Authors:** Ahmed Reda Gonnah, Fahdem Iamrl, Douglas Jacoby, Allison L. Tsao, Mahesh K. Vidula, Daniel E. Soffer, Monika Sanghavi

**Affiliations:** a Department of Internal Medicine, University of Pennsylvania Health System, Philadelphia, Pennsylvania, USA; b Department of Cardiology, University of Pennsylvania Health System, Philadelphia, Pennsylvania, USA

**Keywords:** coronary artery ectasia, inflammation, psoriasis, STEMI, thrombosis

## Abstract

**Background:**

Coronary artery ectasia (CAE) predisposes to abnormal flow, thrombosis, and adverse cardiovascular outcomes.

**Case Summary:**

A 72-year-old woman with diffuse multivessel CAE, systemic arterial ectasia, and psoriasis presented with inferior ST-segment elevation myocardial infarction in the setting of left bundle branch block. Angiography demonstrated an approximately 8-mm ectatic right coronary artery with complete thrombotic occlusion. Aspiration thrombectomy restored partial flow, but stenting was deferred because of diffuse ectasia, persistent thrombus, and malapposition risk. She was discharged on a direct oral anticoagulant plus single antiplatelet therapy.

**Discussion:**

Diffuse CAE is a high-risk thrombotic substrate, and ST-segment elevation myocardial infarction management is challenging because of altered flow, large thrombus burden, and percutaneous coronary intervention limitations.

**Take-Home Message:**

Diffuse systemic ectasia warrants individualized antithrombotic therapy and longitudinal imaging surveillance.

## Visual Summary

**Figure:**
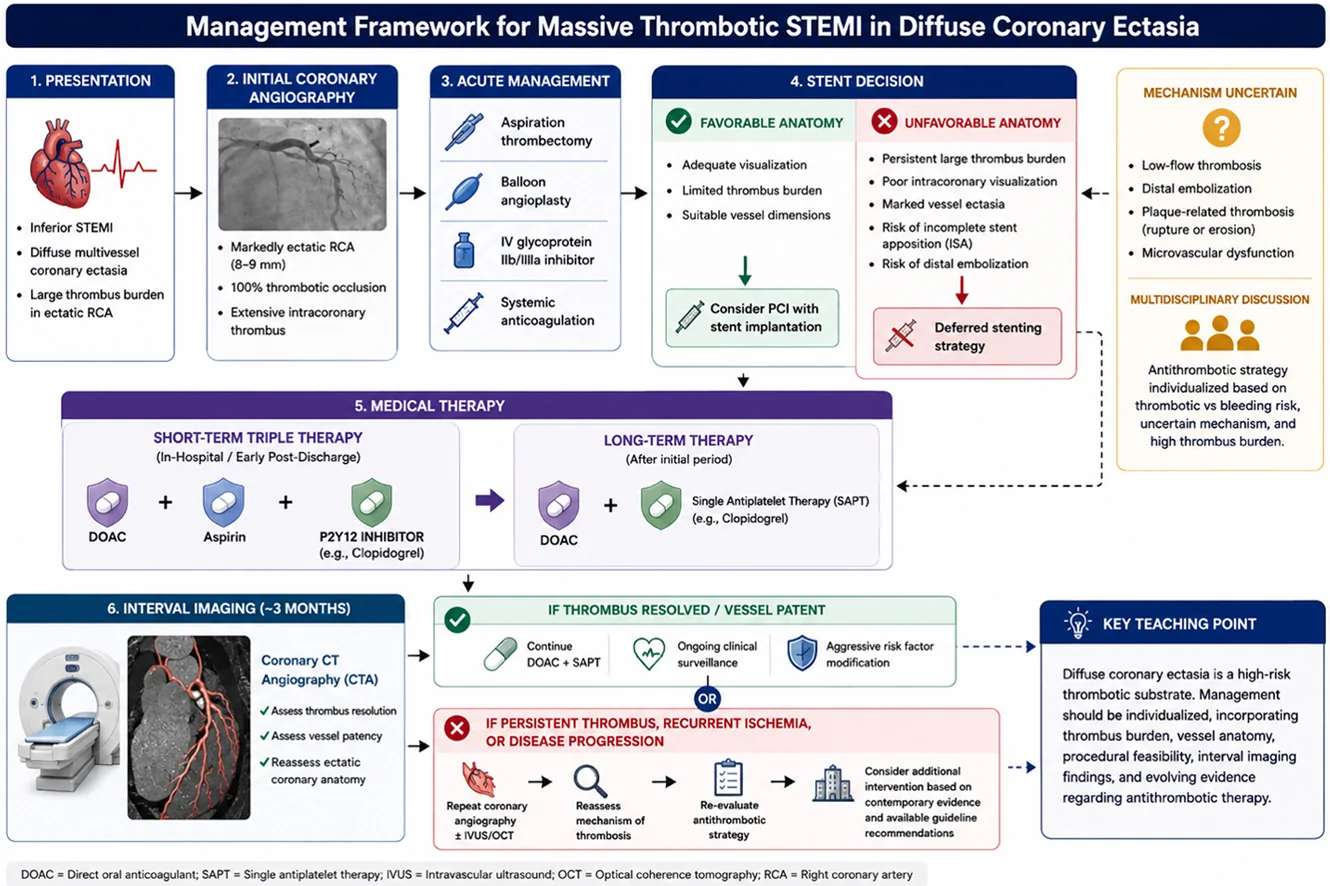

## History and Physical Examination

A 72-year-old woman presented with presyncope while walking. She developed sudden weakness, diaphoresis, arm heaviness, and transient bradycardia detected on her smartwatch. She denied syncope but reported earlier chest discomfort radiating to both upper extremities with diaphoresis. In the emergency department, she was hemodynamically stable (temperature 98.2 °F, blood pressure 97/64 mm Hg, heart rate 64 beats/min, respiratory rate 22 breaths/min, and oxygen saturation 96% on room air). She appeared comfortable and in no acute distress. Cardiovascular examination revealed regular rate and rhythm without murmurs. Lungs were clear to auscultation. Extremities were warm and well perfused without edema. Radial, femoral, and dorsalis pedis pulses were 2+ and symmetric.

## Past Medical History

The patient had known diffuse 3-vessel coronary artery ectasia (CAE) (high-grade type 1 based on Markis Classification of [CAE]) diagnosed in 2022 on coronary computed tomography (CT) angiography (Figure 1), with a coronary artery calcium score of zero and no plaque noted in the coronary arteries. Subsequent imaging demonstrated systemic arterial involvement, including focal superior mesenteric artery aneurysmal dilation and bilateral renal artery ectasia (Figure 2). Prior rheumatologic and genetic evaluations were negative for vasculitis or heritable connective tissue disorders. Additional history included hypertension, hyperlipidemia, prediabetes, prior nonischemic cardiomyopathy with improved ejection fraction, and moderate controlled psoriasis managed with phototherapy. She had no history of Kawasaki disease, smoking, or premature coronary artery disease. She was not receiving any antiplatelet or anticoagulation at the time.

**Figure 1:**
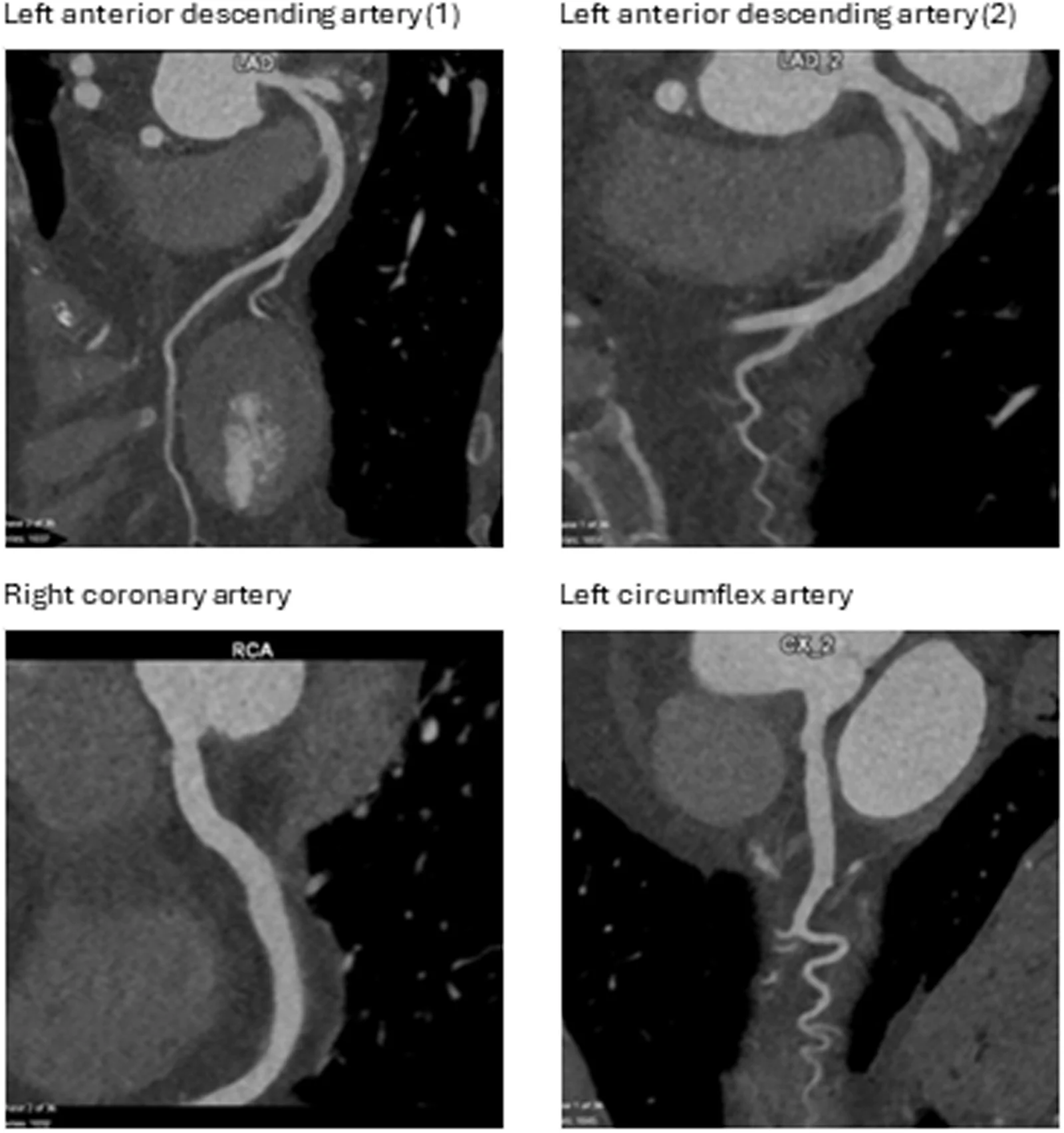
Ectatic Coronary Vessels on Computed Tomography Angiography

**Figure 2:**
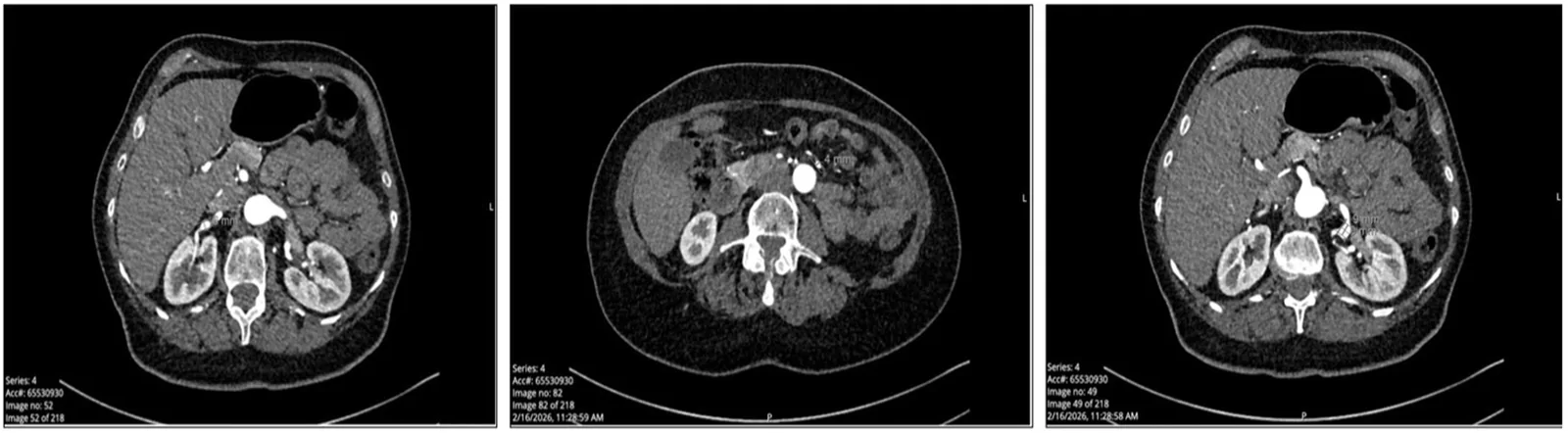
Systemic Arterial Involvement, Including Focal Superior Mesenteric Artery Aneurysmal Dilation and Bilateral Renal Artery Ectasia

## Differential Diagnosis

Differential diagnoses included acute coronary syndrome because of atherothrombosis or thrombosis within an ectatic vessel, coronary embolism, pulmonary embolism, aortic dissection given ascending aortic dilation, and myocarditis.

## Investigations

The initial electrocardiogram showed baseline left bundle branch block with nonspecific ST-segment changes. Serial electrocardiograms revealed inferior ST-segment elevation (leads II and III) with reciprocal changes meeting Sgarbossa Criteria that were not immediately recognized (Figure 3). Troponin rose from normal to 27 ng/L at 1 hour. D-dimer was negative, ruling out pulmonary embolism. Chest radiograph was unremarkable. Transthoracic echocardiography demonstrated a low-normal left ventricular ejection fraction (LVEF) (52%) with new inferior and inferoseptal basal-mid hypokinesis (Video 1) and ascending aorta dilation (4.0 cm). Based on these findings, the patient was diagnosed with ST-segment elevation myocardial infarction (STEMI) and referred for emergent coronary angiography.

**Figure 3:**
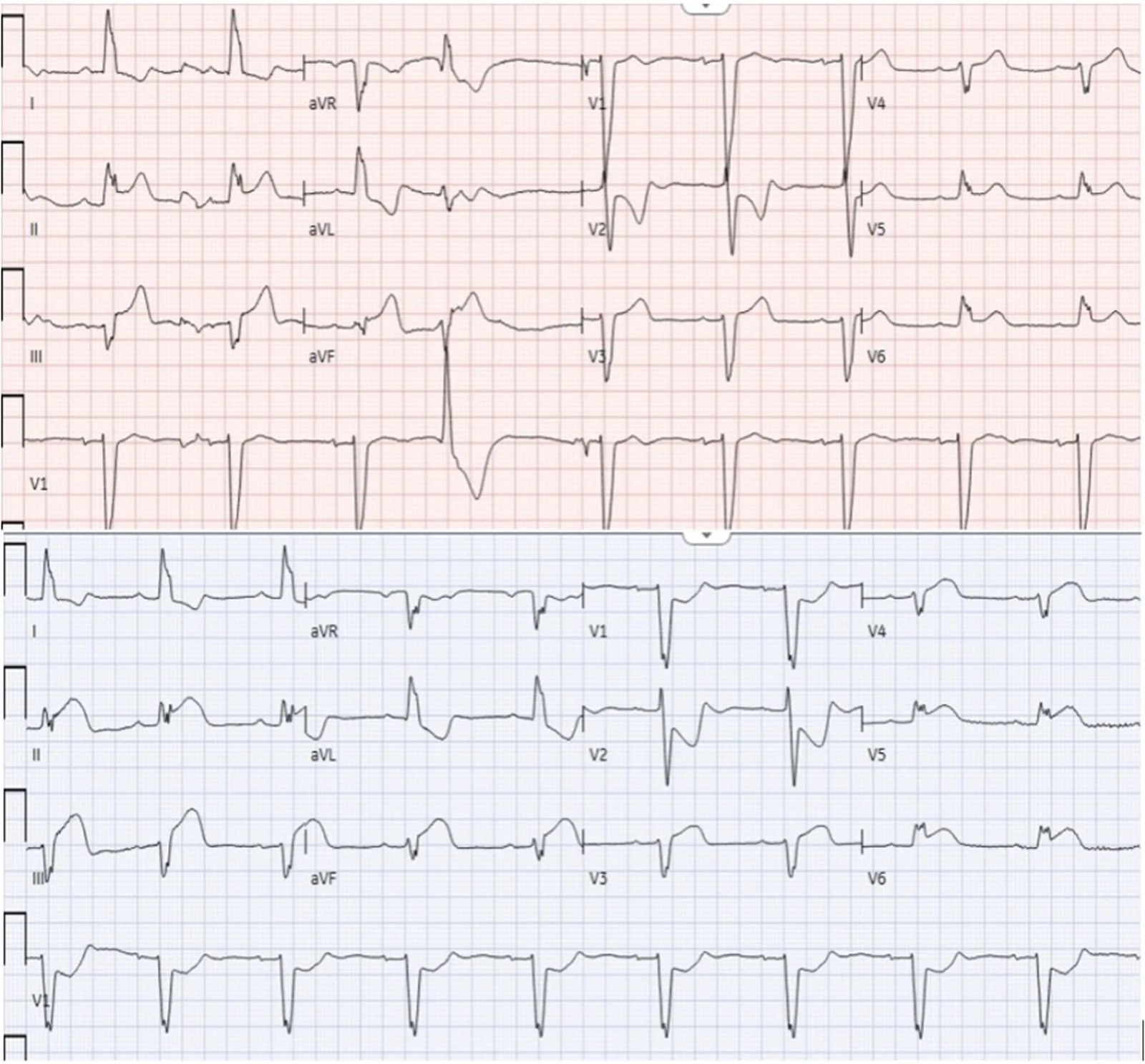
Serial Electrocardiograms From Initial Presentation (Top) and Repeat 1 Hour Later (Bottom)

## Management

The patient was treated emergently for inferior STEMI with aspirin and ticagrelor loading, followed by urgent coronary angiography. Coronary angiography demonstrated diffuse ectasia involving all major epicardial vessels. The left main was large and ectatic without significant stenosis. The left anterior descending artery was ectatic proximally with mild (30%) proximal stenosis and otherwise luminal irregularities. The left circumflex artery was also ectatic with nonobstructive irregularities (Video 2). The right coronary artery (RCA) was dominant, markedly ectatic, and measured approximately 8 × 9 mm on intravascular ultrasound (IVUS), with an upper limit of normal of 4 mm. There was 100% thrombotic occlusion of the mid-RCA. Initial balloon angioplasty resulted in minimal improvement in distal flow. Using a deep-seated guide extender to reduce the risk of systemic embolization, aspiration thrombectomy was performed with improvement to TIMI flow grade 2 in the distal posterior descending artery (Figure 4). Repeat IVUS demonstrated extensive thrombus without clear plaque rupture, although distal evaluation was limited by thrombus burden. Given persistent heavy thrombus burden, diffuse ectasia, and a high risk of stent malapposition, percutaneous stenting was deferred in favor of intensive medical therapy. She was managed with quadruple antithrombotic therapy (dual antiplatelet therapy [DAPT], glycoprotein IIb/IIIa inhibitor, and heparin).

**Figure 4:**
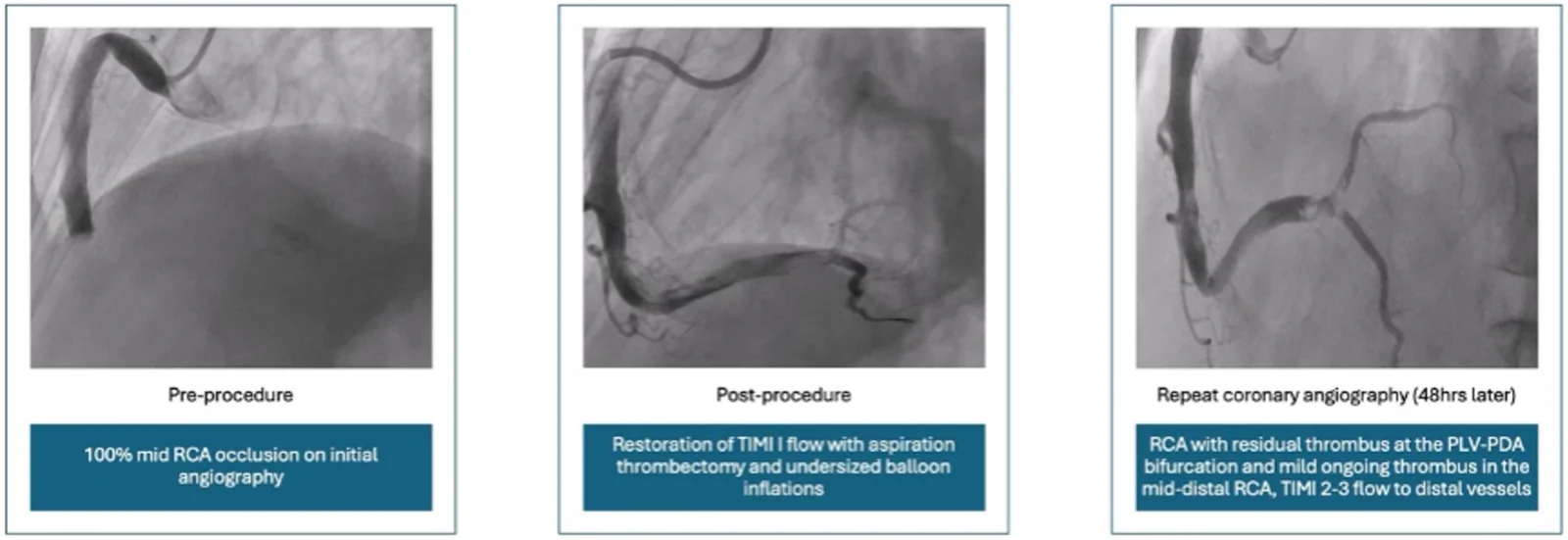
Preprocedural and Postprocedural Coronary Angiography Images Following Mechanical Aspiration Thrombectomy and Balloon Inflations During the Initial Cardiac Catheterization Repeat coronary angiography image from the repeat cardiac catheterization 48 hours later. PLV-PDA = posterior left ventricular-posterior descending artery; RCA = right coronary artery.

Repeat angiography (48 hours later) via 6-F left ulnar access demonstrated residual severe thrombus at the posterolateral ventricular–posterior descending artery bifurcation and mild persistent thrombus in the mid-to-distal RCA. TIMI flow grade 2-3 was present distally. Only mild (10%-20%) mid-RCA irregularity was noted; further IVUS interrogation was deferred because of residual thrombus. The left coronary system was not re-imaged. She was subsequently transitioned to triple therapy with a direct oral anticoagulant (DOAC) plus DAPT until discharge, at which time she was switched to DOAC and single antiplatelet therapy (clopidogrel), along with high-intensity statin, β-blocker, and renin-angiotensin system inhibition.

## Outcome and Follow-Up

The hospitalization was notable for transient peripheral vision changes concerning for an embolic phenomenon; neurologic evaluation revealed low suspicion for acute cerebral infarction, and ophthalmologic assessment was pursued for possible retinal embolism.

She remained hemodynamically stable, with intermittent accelerated idioventricular rhythm but no sustained arrhythmias. LVEF during admission was low-normal at 52%, with elevated N-terminal pro–B-type natriuretic peptide (882 pg/mL). At early outpatient follow-up, she reported excellent symptomatic recovery without angina or heart failure symptoms. Low-density lipoprotein cholesterol was reduced to 45 mg/dL. Her apolipoprotein B was 48 mg/dL. However, repeat echocardiography at 2 months demonstrated progression to mildly reduced systolic function (LVEF 41%) with persistent inferior regional wall motion abnormalities and grade II diastolic dysfunction, consistent with infarct-related remodeling. Ongoing management included continued systemic anticoagulation, single antiplatelet therapy, and guideline-directed medical therapy for left ventricular dysfunction. Repeat CT angiography redemonstrated diffuse ectasia of the coronary arteries and nonobstructive soft plaque in the proximal RCA and left anterior descending with minimal stenosis, with no residual thrombus in the distal bifurcation of the distal RCA into the posterolateral ventricular and posterior descending branches, demonstrating resolution (Figure 5). Her CT fractional flow reserve analysis has shown no evidence of hemodynamically significant stenoses. CT plaque analysis demonstrated a moderate amount of total coronary plaque with a total plaque volume of 268 mm^3^ (the total plaque volume for this patient was at the 72nd percentile for age and sex compared with their peer population).

**Figure 5:**
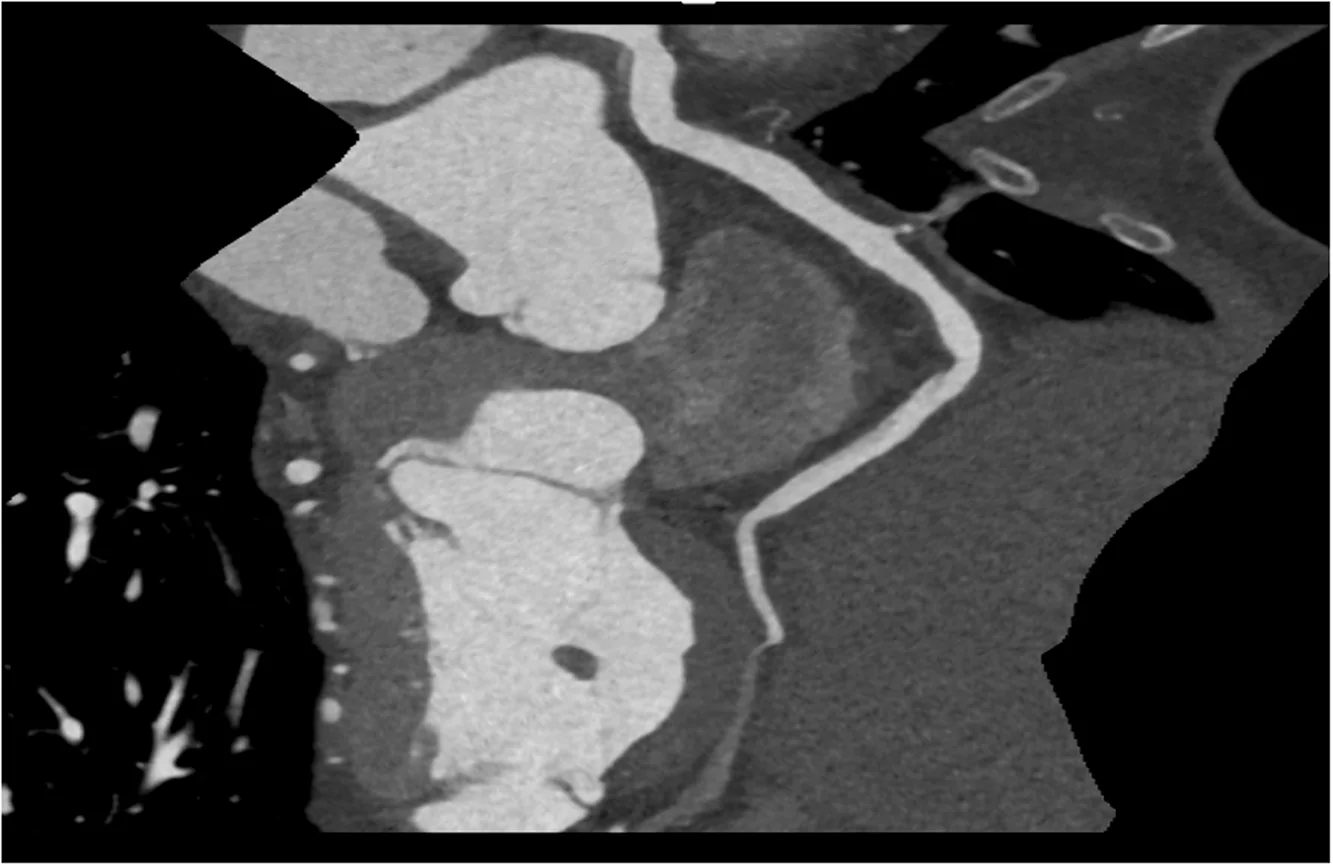
Curved Multiplanar Reconstruction of the Right Coronary Artery, Demonstrating no Evidence of Thrombus

## Discussion

This case illustrates diffuse CAE complicated by massive thrombotic occlusion causing inferior STEMI in the absence of significant obstructive coronary artery disease. The presentation highlights diffuse ectatic coronary disease as a high-risk thrombotic substrate and demonstrates the unique procedural and therapeutic challenges encountered during acute coronary syndromes involving markedly dilated coronary arteries.

CAE, defined as coronary artery dilation ≥1.5 times the diameter of the adjacent normal vessel, is identified in approximately 1%-7% of coronary angiographic studies and has historically been associated with atherosclerosis, although nonatherosclerotic and systemic vascular phenotypes are increasingly recognized.^1^^,^^2^ Histopathologic studies demonstrate medial degeneration, elastin fragmentation, and matrix metalloproteinase dysregulation within ectatic vessels, leading to abnormal vessel wall remodeling and altered coronary hemodynamics.^1^^,^^2^ These structural abnormalities contribute to turbulent flow, delayed contrast clearance, blood stasis, and thrombus formation.^1^, ^2^, ^3^ Importantly, diffuse or high-grade ectasia has been associated with recurrent myocardial infarction and adverse long-term cardiovascular outcomes independent of obstructive coronary disease burden.^3^

In this patient, a markedly ectatic RCA measuring approximately 8-9 mm developed complete thrombotic occlusion despite prior coronary CT angiography demonstrating no coronary calcification or significant plaque burden. The coexistence of coronary, renal, mesenteric, and aortic ectasia further supports an underlying systemic ectatic arteriopathy. This presentation reinforces the concept that thrombosis in CAE may arise from abnormal flow dynamics and local blood stasis within structurally abnormal vessels, although the exact mechanism underlying the STEMI in this case remains uncertain. IVUS demonstrated extensive thrombus burden without clear evidence of plaque rupture; however, definitive exclusion of plaque rupture or plaque erosion was limited by poor intracoronary visualization in the setting of heavy thrombus burden. In addition, there was a delay in recognition related to the baseline left bundle branch block, which may have contributed to progression of thrombus burden before coronary reperfusion. Accordingly, several overlapping mechanisms may have contributed to the presentation, including low-flow thrombosis within the markedly ectatic RCA, plaque-related thrombosis, distal embolization, and downstream microvascular dysfunction. These processes are difficult to fully disentangle in diffuse ectatic coronary disease during acute thrombotic occlusion, particularly when angiographic and intravascular imaging assessments are limited by extensive thrombus burden. We did not pursue intracoronary thrombolytics or glycoprotein IIb/IIIa administration as the patient was chest pain-free with the delayed presentation, and further instrumentation continued to worsen flow. If she had ongoing chest pain, electrical or hemodynamic instability, then that would have been our next step.

Management of acute coronary syndromes in diffuse ectasia presents substantial technical challenges. Large vessel diameter and extensive thrombus burden may reduce the effectiveness of conventional percutaneous coronary intervention techniques and increase the risk of distal embolization. In addition, stent implantation in markedly ectatic vessels carries important concerns regarding stent malapposition, persistent thrombus formation, and late stent thrombosis.^4^ In the present case, aspiration thrombectomy restored partial distal perfusion; however, stent implantation was deferred because of persistent thrombus burden, diffuse dilation, and unfavorable vessel geometry. Because of poor intracoronary visualization in the setting of extensive thrombus burden, implanting a stent with incomplete stent apposition may have created a larger nidus for ongoing thrombus formation. A deferred intervention strategy with intravenous glycoprotein IIb/IIIa inhibitor therapy allowed time for thrombus burden reduction and may have facilitated more precise subsequent coronary intervention if needed. The patient's transient visual symptoms during hospitalization further raised concern for embolic complications related to the extensive intracoronary thrombus burden.

Optimal antithrombotic management in diffuse CAE remains uncertain. Observational data suggest that anticoagulation may reduce thrombotic recurrence in selected patients with diffuse or high-grade ectasia and sluggish coronary flow,^3^ although available evidence remains limited and inconsistent.^5^ More recently, the OVER-TIME (Dual Antiplatelet Therapy Versus Antiplatelet Monotherapy Plus Oral Anticoagulation in Patients With Acute Coronary Syndrome and Coronary Artery Ectasia) trial provided the first randomized evidence in this setting. This open-label, exploratory trial randomized 62 patients with acute coronary syndrome and culprit-vessel coronary artery ectasia to aspirin 100 mg plus clopidogrel 75 mg daily or clopidogrel 75 mg plus rivaroxaban 15 mg daily. At 12 months, the composite of cardiovascular death, recurrent myocardial infarction, or repeat revascularization did not differ significantly between groups (HR: 0.24; 95% CI: 0.02-2.16; *P* = 0.20), although recurrent myocardial infarction occurred numerically less often with clopidogrel plus rivaroxaban (3.3% vs 12.5%). Bleeding rates were similar (HR: 0.75; 95% CI: 0.26-2.16; *P* = 0.59), while fibrin clot lysis time decreased significantly with clopidogrel plus rivaroxaban (−24.7%; *P* = 0.038), but not with DAPT (−14.7%; *P* = 0.25). Given its small sample size, open-label design, and wide confidence intervals, OVER-TIME was underpowered to establish clinical efficacy or safety. Nevertheless, its findings provide mechanistic support for considering individualized anticoagulant-plus-antiplatelet therapy in carefully selected patients with extensive ectatic disease and high thrombotic burden, while awaiting adequately powered trials.^6^

In the present case, there was extensive multidisciplinary discussion regarding the optimal long-term antithrombotic regimen. Given the marked thrombus burden and uncertainty regarding the precise mechanism of thrombosis, the consensus was that the patient should receive both antiplatelet (clopidogrel) and a DOAC. Alternative strategies considered included DAPT alone vs anticoagulation combined with aspirin. However, following a large STEMI, there was preference for maintaining P2Y_12_ inhibition rather than aspirin monotherapy as the antiplatelet backbone. DAPT alone was considered potentially inferior in a patient with diffuse ectasia and massive intracoronary thrombus burden, where abnormal flow dynamics and blood stasis may contribute substantially to thrombogenesis beyond platelet activation alone. Accordingly, the patient was ultimately discharged on a DOAC combined with single antiplatelet therapy using clopidogrel after an initial period of triple therapy during hospitalization. The intended duration of combined therapy will depend on interval reassessment of thrombotic vs bleeding risk, ventricular remodeling, and follow-up coronary imaging, although prolonged anticoagulation is being strongly considered given the diffuse ectatic phenotype and high thrombotic burden observed during presentation.

Psoriasis in this case should be interpreted cautiously as a possible inflammatory cardiovascular risk enhancer rather than a proven mechanistic driver of the patient's diffuse ectatic vasculopathy. Psoriasis is increasingly recognized as a systemic inflammatory condition associated with endothelial dysfunction and elevated cardiovascular risk and is included as a risk-enhancing factor in contemporary prevention guidelines.^7^^,^^8^ Prior reports have suggested potential inflammatory overlap between psoriasis and CAE, including shared cytokine pathways involving tumor necrosis factor-α and interleukin-mediated signaling.^8^^,^^9^ However, evidence directly linking psoriasis to diffuse ectatic arteriopathy remains limited and largely hypothesis-generating. In this patient, the diffuse multiterritorial arterial ectasia likely reflects an underlying systemic vascular phenotype independent of psoriasis itself, although chronic inflammatory signaling may plausibly contribute to endothelial dysfunction or thrombo-inflammatory activation superimposed on this structural substrate. In addition, vascular inflammation and progression of cardiovascular disease may occur despite low clinical inflammatory disease activity in systemic inflammatory disorders.^10^

This case also highlights limitations of traditional atherosclerotic risk assessment tools in patients with atypical vascular phenotypes. Despite a coronary artery calcium score of zero and absence of significant obstructive disease, the patient developed extensive coronary thrombosis and STEMI. Coronary calcium scoring may not adequately capture thrombotic risk related to abnormal vascular remodeling, noncalcified vascular inflammation, or ectatic coronary flow disturbances.

Overall, this case emphasizes diffuse coronary ectasia as a clinically important thrombotic substrate capable of causing life-threatening acute coronary syndromes despite minimal obstructive coronary disease. Further studies are needed to better define optimal surveillance, risk stratification, and antithrombotic management strategies in patients with diffuse ectatic coronary disease.

## Conclusions

Diffuse CAE represents a high-risk vascular phenotype characterized by abnormal coronary flow, thrombus formation, and potentially severe acute coronary syndromes even in the absence of significant obstructive atherosclerosis. This case demonstrates the substantial thrombotic burden and procedural complexity associated with STEMI in markedly ectatic coronary arteries. Although psoriasis may have contributed to systemic inflammatory cardiovascular risk in this patient, its relationship to diffuse ectatic vasculopathy remains uncertain and should be considered hypothesis-generating rather than causal. Greater understanding of thrombotic risk, imaging surveillance, and individualized antithrombotic approaches in diffuse coronary ectasia is needed.

**Visual Summary:**
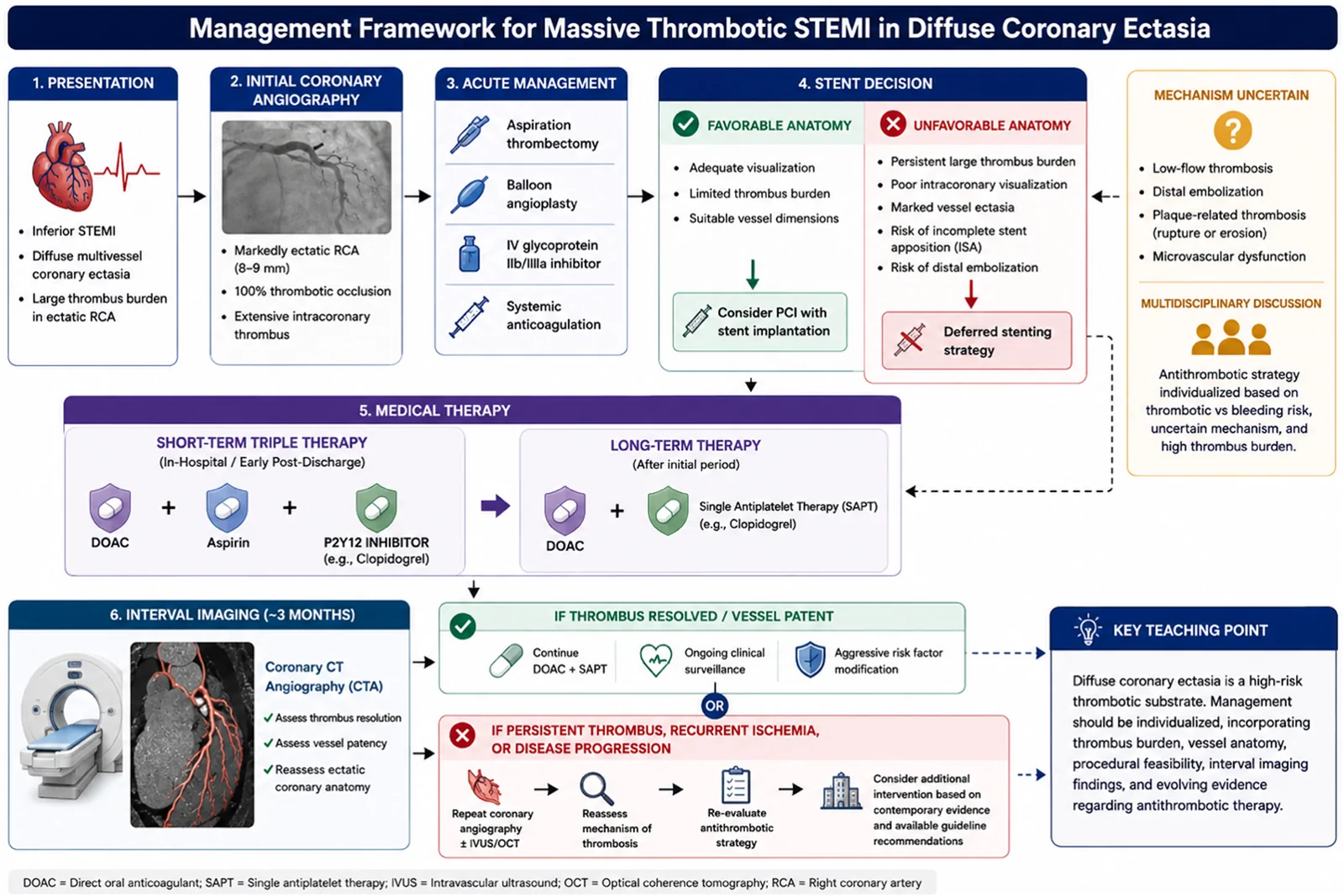
Management Framework of Coronary Artery CTA = computed tomography angiography; DOAC = direct oral anticoagulant; IV = intravenous; IVUS = intravascular ultrasound; OCT = optical coherence tomography; PCI = percutaneous coronary intervention; RCA = right coronary artery; STEMI = ST-segment elevation myocardial infarction.

## Funding Support and Author Disclosures

Dr Soffer has consulting/advisory relationships with Ionis, New Amsterdam, Regeneron, and HeartFlow; investigator relationships with Akcea, Amgen Inc, Amryt, Arrowhead, Ionis, Lilly, Novartis, PCORI, NIH, and HeartFlow; and legal consulting. Dr Jacoby has advisory board relationships with New Amsterdam and Novartis. All other authors have reported that they have no relationships relevant to the contents of this paper to disclose.

**Take-Home Messages**

• Diffuse coronary artery ectasia is a high-risk thrombotic substrate that can cause ST-segment elevation myocardial infarction even without significant obstructive coronary disease.
• Management is challenging because of large thrombus burden, abnormal coronary flow, and technical limitations of percutaneous coronary intervention in markedly ectatic vessels.
• Given the limited evidence available, antithrombotic therapy must be individualized.
• Emerging randomized data suggest that combined antiplatelet and direct oral anticoagulant therapy may be beneficial in selected high-risk patients, although the optimal regimen and duration remain uncertain.

## Appendix

Video 1 Echocardiography Findings Demonstrating New Inferior and Inferoseptal Basal-Mid Hypokinesis on Apical 4-Chamber (A4C) Views (Top) and Parasternal Short-Axis (PSAX) at Mid-Cavity (Bottom)

Video 2 Left Coronary Angiographic Images Demonstrating the Extent of Ectasia in the Left Main, Left Anterior Descending (LAD), and Circumflex Arteries
